# Clinical profiles of treated and untreated adults with hypophosphatasia in the Global HPP Registry

**DOI:** 10.1186/s13023-022-02393-8

**Published:** 2022-07-19

**Authors:** Kathryn M. Dahir, Lothar Seefried, Priya S. Kishnani, Anna Petryk, Wolfgang Högler, Agnès Linglart, Gabriel Ángel Martos-Moreno, Keiichi Ozono, Shona Fang, Cheryl Rockman-Greenberg

**Affiliations:** 1 Program for Metabolic Bone Disorders at Vanderbilt, Endocrinology and Diabetes, Vanderbilt University Medical Center, 8210 Medical Center East, 1215 21st Avenue South, Nashville, TN 37232-8148 USA; 2 University of Würzburg, Würzburg, Germany; 3 Duke University Medical Center, Durham, NC USA; 4 Alexion, AstraZeneca Rare Disease, Boston, MA USA; 5 Department of Paediatrics and Adolescent Medicine, Johannes Kepler University Linz, Linz, Austria; 6 Institute of Metabolism and Systems Research, University of Birmingham, Birmingham, UK; 7 DMU 3 SEA, Service d’endocrinologie et Diabète de L’enfant, filière OSCAR, Centre de Référence des Maladies Rares du Métabolisme du Calcium et du Phosphate, Paris-Saclay University, AP-HP, INSERM, Bicêtre Paris-Saclay Hospital, Le Kremlin-Bicêtre, France; 8 Departments of Pediatrics and Pediatric Endocrinology Hospital Infantil, CIBERobn, ISCIII, Universitario Niño Jesús, IIS La Princesa, Universidad Autónoma de Madrid, Madrid, Spain; 9 Osaka University, Suita, Osaka, Japan; 10 University of Manitoba, Winnipeg, MB Canada

**Keywords:** Hypophosphatasia, Burden of disease, Enzyme replacement therapy, HRQoL

## Abstract

**Background:**

The clinical signs and symptoms of hypophosphatasia (HPP) can manifest during any stage of life. The age at which a patient’s symptoms are reported can impact access to targeted treatment with enzyme replacement therapy (asfotase alfa), as this treatment is indicated for patients with pediatric-onset HPP in most countries. As such, many patients reported to have adult-onset HPP typically do not receive treatment. Comparison of the disease in treated and untreated adult patients is confounded by the approved indication. To avoid this confounding factor, a comparison between baseline disease manifestations prominent among treated versus untreated adult patients was limited to those with pediatric-onset HPP using data collected from the Global HPP Registry. The hypothesis was that treated adults will have a greater disease burden at baseline than untreated adults. The analysis of disease manifestations in adults with adult-onset HPP was conducted separately.

**Results:**

A total of 398 adults with HPP were included; 213 with pediatric-onset (114 treated, 99 untreated) and 141 with adult-onset HPP (2 treated and 139 untreated). The treated, pediatric-onset patients were more likely to have a history of pain (prevalence ratio [PR]: 1.3, 95% confidence interval [CI] 1.1, 1.4), skeletal (PR: 1.3, 95% CI 1.1, 1.6), constitutional/metabolic (PR: 1.7, 95% CI 1.3, 2.0), muscular (PR: 1.8, 95% CI 1.4, 2.1) and neurological (PR: 1.7, 95% CI 1.1, 2.3) manifestations of HPP, and also had poorer measures for health-related quality of life, pain, and disability compared with untreated pediatric-onset patients. In patients with adult-onset HPP, the most frequent signs and symptoms were chronic bone pain (52.5%), dental manifestations (42.6%), fatigue (23.4%), recurrent fractures or pseudofractures (22.0%), and generalized body pain (22.0%).

**Conclusions:**

Along with the more classical skeletal signs and symptoms, pain, muscular, and constitutional/metabolic manifestations are common in adults with HPP, regardless of age of disease onset, highlighting a full spectrum of HPP manifestations.

## Introduction

Hypophosphatasia (HPP) is a rare, inherited disorder associated with low alkaline phosphatase (ALP) due to tissue-nonspecific alkaline phosphatase (TNSALP) deficiency resulting from > 400 genetic aberrations in *ALPL* [1, 2]. Deficiency of TNSALP leads to elevated serum levels of its endogenous substrates, namely inorganic pyrophosphate (PPi), pyridoxal 5‘ phosphate (PLP), and phosphoethanolamine (PEA), a component of the phosphatidylinositol-glycan linkage apparatus that couples TNSALP and other proteins to plasma membranes [3, 4]. This deficit of TNSALP activity can lead to skeletal hypomineralization and nonskeletal manifestations affecting multiple body systems. Notably, these signs and symptoms can have a significant effect on health-related quality of life (HRQoL) in adults with HPP, especially relating to pain and physical function [5, 6].

Some adults with HPP may experience a lifetime of HPP manifestations, sometimes beginning in utero or early childhood (pediatric-onset HPP, age < 18 years) [7], whereas in others, the manifestations of HPP become apparent only later in life (adult-onset HPP, age ≥ 18 years) [5, 7]. Previous analyses from the Global HPP Registry have shown that regardless of age of disease onset, adults with HPP have a high disease burden that negatively impacts patient-reported HRQoL [8].

The current indication for treatment of HPP with enzyme replacement therapy (ERT) is limited to adults with pediatric-onset HPP, with the exception of Japan, where there is no limitation for ERT by age of onset, and this may elicit a recall bias towards retrospective reporting of childhood symptoms in adult patients.

Irrespective of the age of onset, the variable clinical presentation of HPP in adults often leads to misdiagnosis, substantial delays in diagnosis, delays in treatment and multidisciplinary supportive care (e.g., genetic counseling, physical therapy, pain management, occupational therapy, and dental care), or exposure to potential harmful treatment, such as bisphosphonates [8–10]. To better inform disease management, this study aimed to understand the clinical profiles of adults with HPP. Baseline HPP manifestations were analyzed by treatment status using data from the Global HPP Registry. This analysis only included adults with documented pediatric-onset HPP to avoid potential bias from the current indications for ERT in most countries, which exclude adult-onset HPP. In addition, HRQoL, pain, and disability were assessed for those with pediatric-onset HPP. To fully characterize the clinical profiles of HPP in adults, burden of illness was also analyzed in adults with reported adult-onset HPP.

## Methods

### Study design and data source

A retrospective, observational study was conducted using data from the Global HPP Registry collected through September 7, 2020. The Global HPP Registry design and data collection have been previously described by Högler et al. [11]. To summarize, at enrollment, physicians provided data on baseline demographics and clinical characteristics of HPP including medical history, manifestations of HPP, treatment status, age at the first HPP manifestation, and the results of patient-reported outcome (PRO) measures from consenting participants collected during routine clinical practice.

The Global HPP Registry was initiated in 2015 as an observational, prospective, multinational study of patients with HPP (NCT02306720; EUPAS13526). The registry is sponsored by Alexion, AstraZeneca Rare Disease (Boston, MA, USA) and is overseen by a scientific advisory board comprising clinical experts in HPP and stakeholders at Alexion, AstraZeneca Rare Disease [8, 11].

### Patient population

In this study, adults (aged ≥ 18 years at baseline) with a clinical diagnosis of HPP based on a low serum ALP activity below the age- and sex-adjusted reference intervals and/or at least one *ALPL* variant were eligible for inclusion. For untreated patients (those who were managed without ERT), data from the time of registry enrollment were analyzed. For treated patients (those who went on to receive ERT), data from before the start of treatment with ERT were analyzed. Patients were also required to have data on enrollment date, date of birth, sex, treatment status (including start date if initiated on ERT), and a record of at least one sign or symptom of HPP.

### Outcomes

Demographics, clinical characteristics of HPP, medical history related to HPP manifestations, and PRO measures were assessed in the overall population, by age of onset, and by treatment status. PRO measures included the 36-item Short Form Health Survey version 2 (SF-36v2), the Brief Pain Inventory Short Form (BPI-SF), and the Health Assessment Questionnaire and Disability Index (HAQ-DI).

### Statistical analysis

SAS Life Science Analytics Framework 5.2.1 was used for all statistical analyses. Descriptive statistics were calculated for the baseline demographics and clinical characteristics of HPP, including HPP manifestations and PRO measures, at baseline. Continuous variables were reported using median (minimum [min], maximum [max]) and mean (standard deviation [SD]), as appropriate. Categorical variables were reported as number (n), percent. To understand the relative frequency of HPP manifestations in treated compared with untreated adults with pediatric-onset HPP, prevalence ratios (PRs) and 95% confidence intervals (CIs) were calculated. PRs were calculated as the ratio of the proportion in the treated group to the proportion in the untreated group; 95% CIs were calculated using the binomial approximation. In addition, among those with pediatric-onset HPP, BPI-SF and HAQ-DI scores were compared between treated and untreated adults using the Wilcoxon rank sum test; SF-36v2 scores were compared for treated versus untreated adults using a two-sample *t*-test. For the SF-36v2, norm-based physical component summary (PCS) and mental component summary (MCS) scores were derived from the individual item responses with the Quality Metrics Health Outcome Scoring Software 5.0 [12, 13]. Mean PCS and MCS scores were compared with the 1998 United States (US) general adult population norm (mean: 50; SD: 10) as previously described by Ware et al. [13] and in a recent study by Seefried and colleagues [8]. Statistical significance was defined as *P* < 0.05.

## Results

This analysis aimed to compare baseline HPP manifestations between treated and untreated adults with pediatric-onset HPP because all patients with pediatric-onset HPP are eligible to receive treatment with asfotase alfa. Therefore, adults in the Global HPP Registry were divided by age of onset (adult versus pediatric-onset HPP).

### Baseline patient characteristics

Of the patients in the Global HPP Registry, 398 adults met the inclusion criteria for this analysis. Demographics and clinical characteristics of HPP for the overall population and for those stratified by age of onset (pediatric-onset HPP and adult-onset HPP) are reported in Table 1. In the overall population, most adults were women (72.6%), and the median age at first manifestation was 14.4 years (min, max: 0.0, 75.3 years). The median age at HPP diagnosis in the overall population was 42.3 years (min, max: 0.0, 78.9).

**Table 1:** Baseline demographics and clinical characteristics of HPP in adults (aged ≥ 18 years)

Characteristic	Adults (overall)^a^ N = 398	Pediatric-onset HPP n = 213	Adult-onset HPP n = 141
*Sex, n (%)*			
Male	109 (27.4)	68 (31.9%)	34 (24.1)
Female	289 (72.6)	145 (68.1%)	107 (75.9)
*Age at baseline, years*			
Mean (SD) Median (minimum, maximum)	47.5 (14.9) 48.1 (18.3, 81.2)	44.8 (15.1) 43.8 (18.3, 77.9)	51.4 (13.9) 51.9 (19.5, 79.8)
*Age at first HPP manifestation, years*			
n Mean (SD)	275 22.1 (20.9)	161 8.6 (11.5)	114 41.2 (15.6)
Median (minimum, maximum)	14.4 (0.0, 75.3)	5.0 (0.0, 72.0)	39.0 (18.0, 75.3)
*Age at HPP diagnosis, years*			
n Mean (SD) Median (minimum, maximum)	349 41.3 (19.3) 42.3 (0.0, 78.9)	184 35.2 (21.0) 36.0 (0.0, 77.0)	131 48.9 (13.9) 49.0 (18.7, 76.6)
*Received ERT, n (%)*	119 (29.9%)	114 (53.5%)	2 (1.4%)
*Baseline ALP, (U/L)*^b^			
n Mean (SD) Median (minimum, maximum)	344 26.4 (13.8) 24.0 (2.0, 98.0)	176 25.1 (15.0) 22.5 (2.0, 98.0)	128 27.5 (12.8) 25.0 (7.0, 98.0)

*ALP* Alkaline phosphatase, *ERT* enzyme replacement therapy, *HPP* hypophosphatasia, *SD* standard deviation

^a^Includes 44 patients with missing or unknown age of onset

^b^Measures may have been obtained during adulthood (age ≥ 18 years) or childhood (age < 18 years)

About half of the patients in the pediatric-onset group went on to ERT (53.5%), whereas only 1.4% of patients with adult-onset HPP went on to ERT. Regardless of age of onset, all 344 adults with data on serum ALP had levels below the age- and sex-adjusted normal range.

### Treated adult patients with pediatric-onset HPP have a higher skeletal and nonskeletal disease burden at baseline than those with pediatric onset who were untreated

**Fig. 1:**
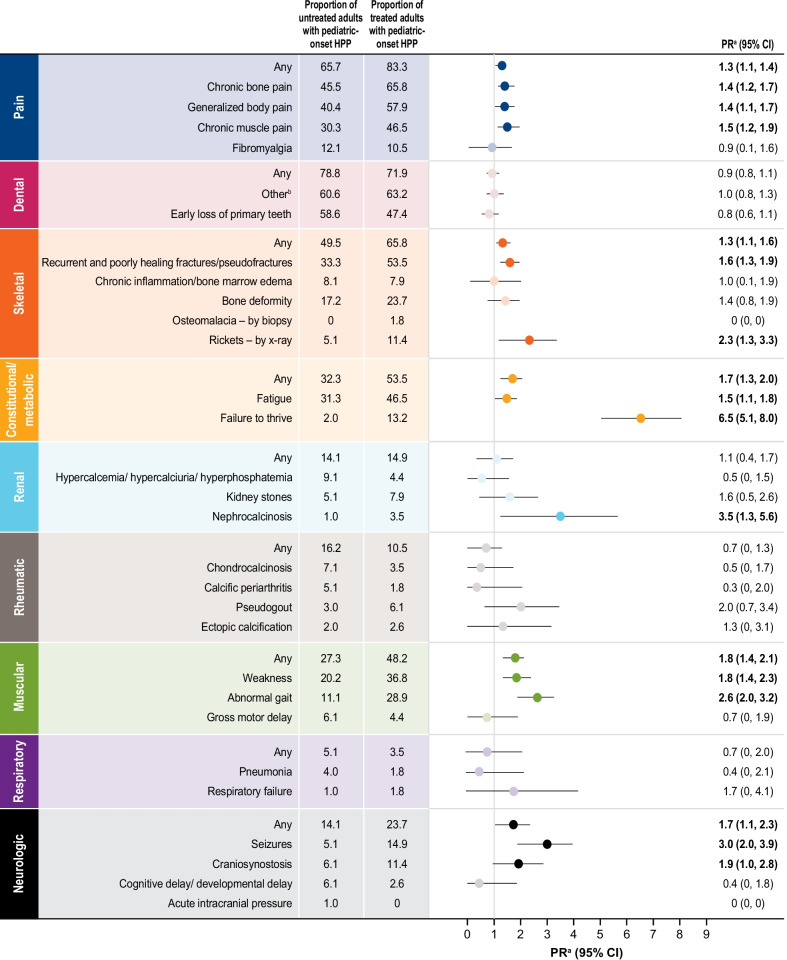
History of HPP manifestations at baseline among treated (n = 99) and untreated (n = 114) adults with pediatric-onset HPP. ^a^PR of treated adults/untreated adults with pediatric-onset HPP. PR > 1 represents an increased likelihood that treated patients had a history of the indicated HPP manifestation. Numbers in bold represent PRs with 95% CI values > 1. ^b^Includes loss of permanent teeth, loose teeth, poor dentition, hypodontia, dental implants, dental bridges, and dentures. *CI*, confidence interval; *HPP*, hypophosphatasia; *PR*, prevalence ratio

To better understand the signs and symptoms of HPP in those with pediatric-onset HPP, the clinical profiles of adults with pediatric-onset HPP were compared based on treatment status (with ERT versus without ERT). Those who went on to ERT were more likely to have a history of pain (83.3% versus 65.7%; PR: 1.3, 95% CI: 1.1, 1.4), skeletal (65.8% vs. 49.5%; PR: 1.3, 95% CI: 1.1, 1.6), constitutional/metabolic (53.5% versus 32.3%; PR: 1.7, 95% CI: 1.3, 2.0), muscular (48.2% versus 27.3%; PR: 1.8, 95% CI: 1.4, 2.1), and neurological (23.7% vs. 14.1%; PR: 1.7, 95% CI: 1.1, 2.3), manifestations of HPP than those who were managed without ERT (Fig. 1). A high proportion of both treated and untreated patients had skeletal (65.8% vs. 49.5%), dental (71.9% vs. 78.8%), and pain (83.3% vs. 65.7%) manifestations of HPP at baseline (Fig. 1).

When analyzing the specific history of signs and symptoms of HPP by treatment status, the largest differences observed between treated and untreated patients were rickets—by X-ray (11.4% versus 5.1%; PR: 2.3, 95% CI: 1.3, 3.3), abnormal gait (28.9% vs. 11.1%; PR: 2.6, 95% CI: 2.0, 3.2), seizures (14.9% vs. 5.1%; PR: 3.0, 95% CI: 2.0, 3.9), nephrocalcinosis (3.5% vs. 1.0%; PR: 3.5, 95% CI: 1.3, 5.6), and failure to thrive (13.2% vs. 2.0%; PR: 6.5, 95% CI: 5.1, 8.0; Fig. 1). Histories of craniosynostosis, muscular weakness, recurrent and poorly healing fractures/pseudofractures, chronic muscle pain, fatigue, chronic bone pain, and generalized body pain were also noticeably more common at baseline among those who went on to be treated with ERT compared with those who did not receive ERT (Fig. 1).

### Patients with pediatric-onset HPP who went on to ERT had poorer HRQoL, pain, and disability outcomes at baseline than untreated pediatric onset patients

**Fig. 2:**
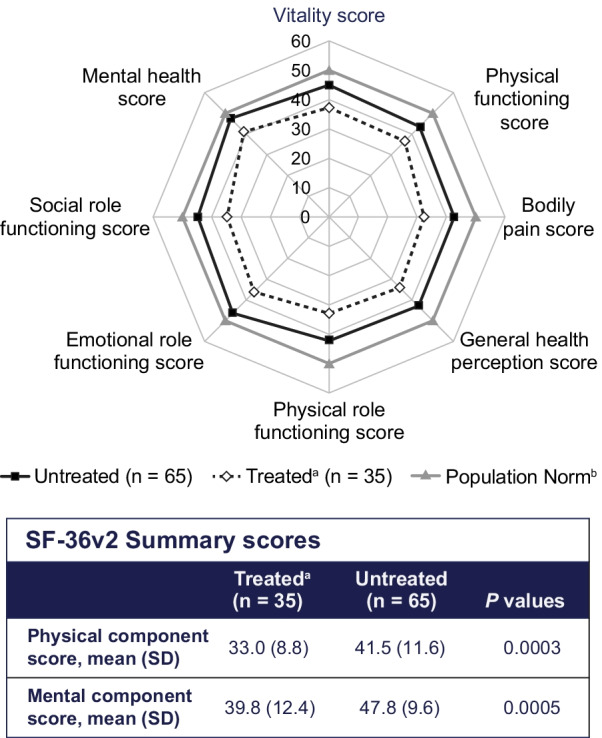
Summary of mean SF-36v2 scores at baseline among treated and untreated adults with pediatric-onset HPP. ^a^Before the start of ERT. ^b^Based on normative data reported in Ware et al.^3^ for the 1998 US general population. *ERT*, enzyme replacement therapy; *HPP*, hypophosphatasia; *HRQoL*, health-related quality of life; *SD*, standard deviation; *SF-36v2*, 36-item Short Form Health Survey version 2; *US*, United States

**Fig. 3:**
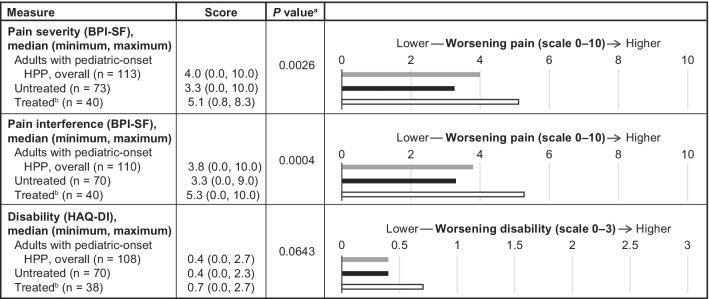
Pain and disability at baseline among adults with pediatric-onset HPP: overall and by treatment status. ^a^Comparison between untreated and treated. ^b^Before the start of ERT. *BPI-SF*, Brief Pain Inventory Short Form; *ERT*, enzyme replacement therapy; *HAQ-DI*, Health Assessment Questionnaire Disability Index; *HPP*, hypophosphatasia

The impact of HPP on HRQoL, pain, and disability was compared only between treated and untreated adults with pediatric-onset HPP. These analyses have previously been reported in adults with adult-onset disease [8]. Results of the PRO measures showed that both treated and untreated adults with pediatric-onset HPP had lower (worse) scores in all eight domains (physical functioning, physical role limitations, bodily pain, general health perceptions, vitality, social functioning, emotional role limitations, and mental health) of the SF-36v2 when compared with normative data from the US general population (Fig. 2). At baseline, treated adults with pediatric-onset HPP had lower scores than those who were untreated in all eight domains of the SF-36v2, and both PCS (*P* = 0.0003) and MCS scores (*P* = 0.0005) for the SF-36v2 showed that baseline HRQoL for treated adults was lower than for untreated adults (Fig. 2). Notably, these differences were observed despite the small number of treated patients with available data. In addition, treated adults with pediatric-onset HPP reported greater pain severity via the BPI-SF (*P* = 0.0026) and pain interference via the BPI-SF (*P* = 0.0004) and a similar level of disability (*P* = 0.0643) via the HAQ-DI when compared with untreated adults with pediatric-onset HPP (Fig. 3).

### Many patients with adult-onset HPP experience substantial disease burden

**Fig. 4:**
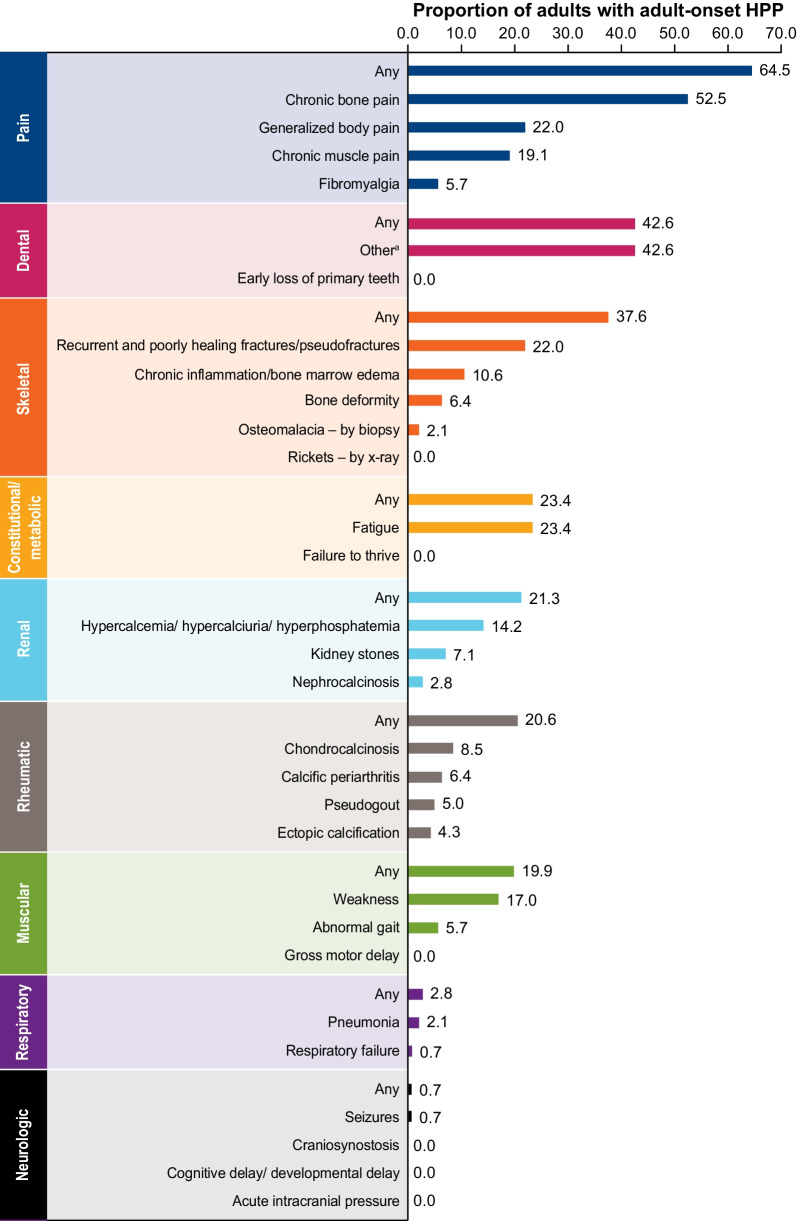
History of HPP manifestations at baseline among adults with adult-onset HPP (n = 141). ^a^Includes loss of permanent teeth, loose teeth, poor dentition, hypodontia, dental implants, dental bridges, and dentures. *HPP*, hypophosphatasia

A thorough understanding of HPP in adults requires an analysis of HPP manifestations in those who report their first HPP manifestations in adulthood because a substantial burden of illness can also be present in this group [8]. The baseline disease burden in patients with adult-onset HPP is presented in Fig. 4. A history of pain (64.5%), dental (42.6%), skeletal (37.6%), constitutional/metabolic (23.4%), and renal (21.3%) manifestations were most common among this population. Chronic bone pain (52.5%) and dental manifestations such as loss of permanent teeth, loose teeth, and poor dentition (42.6%) were the most frequently recorded manifestations of HPP in these adults. Recurrent and poorly healing fractures and/or pseudofractures were the most frequently reported skeletal manifestation (22.0%). A history of chronic inflammation and bone marrow edema, which might represent stress reactions, was reported in 10.6% of these adults. Fatigue was a frequent symptom reported in about 23.4% of adults. Generalized body pain and chronic muscle pain were also reported in 22.0% and 19.1% of adults, respectively.

## Discussion

Results of this study show that adults with HPP are broadly affected by both skeletal and nonskeletal manifestations, regardless of age of HPP onset. Importantly, this study adds evidence to the growing body of literature showing that nonskeletal manifestations commonly occur in a disease that has been classically considered to mostly effect the skeletal system [5, 8, 11, 14–16]. Specifically, results of this analysis identified a higher prevalence of muscular and constitutional/metabolic manifestations in adults with pediatric-onset HPP who went on to ERT than those who were managed without ERT. Although the etiology of muscle weakness and fatigue in HPP is poorly understood, TNSALP deficiency is associated with elevated PLP levels, mitochondrial hyperfunction, diminished neural function, and high adenosine triphosphate (ATP) levels, which have been posited to cause muscle weakness and/or fatigue by impairing energy metabolism [17, 18]. Additionally, muscle weakness could be due to the excess extracellular inorganic PPi characteristic of HPP [19–21]. Although it may be at least partially related to the underlying bone pathology, more studies are needed to understand the role of TNSALP in energy metabolism and muscle function.

Results of this study also show that treated adults with pediatric-onset HPP had a higher prevalence of pain, skeletal, and neurological manifestations of HPP than untreated adults with pediatric-onset HPP. However, a high proportion of both treated and untreated adults with pediatric-onset HPP had skeletal, dental, and pain manifestations at baseline.

In this analysis, the manifestations experienced by adult-onset patients are noticeably similar to pediatric-onset patients who went on to ERT. Therefore, substantial disease burden still exists in patients with adult-onset HPP, which may be the result of underreporting of symptoms in childhood. Underreporting of pediatric symptoms may occur because of poor health literacy and access to care, but also due to historically decreased awareness for less critical manifestations in contemporary adults. This represents a potential gap in patient care, which is supported by the finding that treated adults were almost entirely reported to have pediatric-onset HPP, and only 1.4% of patients with adult-onset HPP went on to ERT despite the substantial disease burden. The current regulatory approval of ERT only for patients with pediatric-onset HPP (except for Japan) most likely accounts for the disparity in treatment by age of onset.

Adolescents and adults with documented pediatric-onset HPP benefit from ERT by experiencing reduced pain, improved mobility, and muscle strength [22]. Magdaleno et al. reported that ERT may provide benefit for adult-onset HPP in reducing pain, improving mobility, and healing fractures [23]. Other reports showed that ERT was considered for patients with adult-onset HPP, but access issues required the use of alternate, less targeted therapeutic options [24–26].

Because the manifestations of HPP have previously been shown to have a significant effect on PRO measures for HRQoL, pain, and disability [5, 8], these outcomes were also assessed in this study based on treatment status. Results of this study show that both treated and untreated adults with HPP experience pain, disability, and reduced HRQoL. However, HRQoL outcomes and pain were worse in treated adults at baseline, suggesting that those who went on to ERT experienced more severe disease.

### Limitations

Findings of this study are subject to some limitations. Because data were collected and entered in the registry by physicians during routine clinical practice, differences in the standard of care from site to site may have led to variations in the availability of data. Although the data used in this study were obtained from medical records overseen by a physician, characteristics such as age at first HPP manifestation, age at HPP diagnosis, history of HPP manifestations, and PRO measures may have been subject to recall bias resulting from insufficient patient memory if they were reported to the physician by the patient. In addition, the long recall times between an incident and when a patient recalled it, which may have been required of some adult patients, could have reduced the accuracy of the data.

Prior studies have shown that there can be phenotypic overlap between HPP onset types and that some patients who are diagnosed with adult-onset HPP truly have unrecognized pediatric-onset HPP [5, 14, 27]. In addition, although life-threatening disease may be more easily recognized at an early age, the totality of HPP manifestations that appear later in life may accrue and evolve over time [16, 28].

## Conclusions

In this analysis of the Global HPP Registry, adults with HPP had a broad history of both skeletal and nonskeletal manifestations of HPP. Patients with adult-onset HPP experienced many disease manifestations that are shared with patients with pediatric-onset disease who went on to ERT. At baseline, both treated and untreated adults with pediatric-onset HPP had a high prevalence of pain, dental, and skeletal manifestations; however, the group of patients who went on to treatment with ERT exhibited more manifestations of HPP and poorer PRO measures for HRQoL and pain at baseline than untreated adults. These findings highlight the need for earlier diagnosis of HPP, better identification of treatable clinical burden, and the screening of family members of patients with HPP. In addition, future studies that correlate genotype/phenotype data with the clinical profiles of adults with HPP may also help to inform more effective disease management decisions.

## Data Availability

Alexion, AstraZeneca Rare Disease will consider requests for disclosure of clinical study participant-level data provided that participant privacy is assured through methods such as data de-identification, pseudonymization, or anonymization (as required by applicable law), and if such disclosure was included in the relevant study informed consent form or similar documentation. Qualified academic investigators may request participant-level clinical data and supporting documents (statistical analysis plan and protocol) pertaining to Alexion-sponsored studies. Further details regarding data availability and instructions for requesting information are available in the Alexion Clinical Trials Disclosure and Transparency Policy at https://alexion.com/our-research/research-and-development. Link to Data Request Form (https://alexion.com/contact-alexion/medical-information).

## Abbreviations

ALP: Alkaline phosphatase
ATP: Adenosine triphosphate
BPI-SF: Brief Pain Inventory Short Form
CI: Confidence interval
ERT: Enzyme replacement therapy
EU: European Union
HAQ-DI: Health Assessment Questionnaire and Disability Index
HPP: Hypophosphatasia
HRQoL: Health-related quality of life
MCS: Mental component summary
PCS: Physical component summary
PEA: Phosphoethanolamine
PLP: Pyridoxal 5’ phosphate
PR: Prevalence ratio
PRO: Patient-reported outcome
SD: Standard deviation
SF-36v2: 36-Item Short Form Health Survey version 2
TNSALP: Tissue-nonspecific alkaline phosphatase
US: United States
